# Social norms and beliefs about gender based violence scale: a measure for use with gender based violence prevention programs in low-resource and humanitarian settings

**DOI:** 10.1186/s13031-019-0189-x

**Published:** 2019-03-08

**Authors:** Nancy Perrin, Mendy Marsh, Amber Clough, Amelie Desgroppes, Clement Yope Phanuel, Ali Abdi, Francesco Kaburu, Silje Heitmann, Masumi Yamashina, Brendan Ross, Sophie Read-Hamilton, Rachael Turner, Lori Heise, Nancy Glass

**Affiliations:** 1 Johns Hopkins School of Nursing, 525 North Wolfe Street, Baltimore, MD 21205 USA; 2 UNICEF, New York, NY USA; 3 Comitato Internazionale per lo Sviluppo dei Popoli (CISP) Somalia, Nairobi, Kenya; 4 Voice For Change, Yei, South Sudan; 5 Norwegian Church Aid, Oslo, Norway; 6 UNICEF, Geneva, Switzerland; 7 UNICEF Somalia, Mogadishu, Somalia; 8 Consultant, Gender based violence in Emergencies, Sydney, Australia; 9 Johns Hopkins Bloomberg School of Public Health, Baltimore, USA

**Keywords:** Gender-based violence, Global health, Humanitarian, Metrics, Scale, Social norms

## Abstract

**Background:**

Gender-based violence (GBV) primary prevention programs seek to facilitate change by addressing the underlying causes and drivers of violence against women and girls at a population level. Social norms are contextually and socially derived collective expectations of appropriate behaviors. Harmful social norms that sustain GBV include women’s sexual purity, protecting family honor over women’s safety, and men’s authority to discipline women and children. To evaluate the impact of GBV prevention programs, our team sought to develop a brief, valid, and reliable measure to examine change over time in harmful social norms and personal beliefs that maintain and tolerate sexual violence and other forms of GBV against women and girls in low resource and complex humanitarian settings.

**Methods:**

The development and testing of the scale was conducted in two phases: 1) formative phase of qualitative inquiry to identify social norms and personal beliefs that sustain and justify GBV perpetration against women and girls; and 2) testing phase using quantitative methods to conduct a psychometric evaluation of the new scale in targeted areas of Somalia and South Sudan.

**Results:**

The *Social Norms and Beliefs about GBV Scale* was administered to 602 randomly selected men (*N* = 301) and women (N = 301) community members age 15 years and older across Mogadishu, Somalia and Yei and Warrup, South Sudan. The psychometric properties of the 30-item scale are strong. Each of the three subscales, “Response to Sexual Violence,” “Protecting Family Honor,” and “Husband’s Right to Use Violence” within the two domains, personal beliefs and injunctive social norms, illustrate good factor structure, acceptable internal consistency, reliability, and are supported by the significance of the hypothesized group differences.

**Conclusions:**

We encourage and recommend that researchers and practitioners apply the *Social Norms and Beliefs about GBV Scale* in different humanitarian and global LMIC settings and collect parallel data on a range of GBV outcomes. This will allow us to further validate the scale by triangulating its findings with GBV experiences and perpetration and assess its generalizability across diverse settings.

**Electronic supplementary material:**

The online version of this article (10.1186/s13031-019-0189-x) contains supplementary material, which is available to authorized users.

## Introduction

Gender-based violence (GBV) remains one of the most prevalent and persistent issues facing women and girls globally [1–4]. Conflict and other humanitarian emergencies place women and girls at increased risk of many forms of GBV [5–7]. The Inter-Agency Standing Committee (IASC) 2015 *Guidelines for Integrating GBV Interventions in Humanitarian Action* defines GBV as any harmful act that is perpetrated against a person’s will and that is based on socially ascribed (i.e., gender) differences between females and males. It includes acts that inflict physical, sexual or mental harm or suffering, threats of such acts, coercion, and other deprivations of liberty. These harmful acts can occur in public and in private [8]. There continues to be limited global information on the burden of GBV in humanitarian emergencies. One systematic review found that approximately one in five refugees or displaced women in complex humanitarian settings experienced sexual violence, though this is likely an underestimation of the true prevalence given the many barriers to survivors’ disclosure of GBV [9]. A recent population-based survey on GBV across the three regions of Somalia examined typology and scope of GBV victimization with 2376 women (15 years and older). The study found that among women, 35.6% (95% CI 33.4 to 37.9) reported lifetime experiences of physical or sexual intimate partner violence (IPV) and 16.5% (95% CI 15.1 to 18.1) reported lifetime experience of physical or sexual non-partner violence (NPV) since the age of 15 years. Women at greatest risk of GBV (IPV and NPV) included membership in a minority clan, displacement from home because of conflict or natural disaster, husband/partner use of khat (e.g., leaves chewed or drunk as a stimulant), exposure to parental violence and violence during childhood. Women survivors of GBV consistently report negative impacts on physical, mental and reproductive health. Often negative health and social consequences are never addressed because women do not disclose GBV to providers or access health care or other services (e.g., protection, legal, traditional authorities) because of social norms that blame the woman for the assault (e.g., she was out alone after dark, she was not modestly dressed, she is working outside the home), norms that prioritize protecting family honor over safety of the survivor, and institutional acceptance of GBV as a normal and expected part of displacement and conflict [10–13].

### GBV primary prevention in humanitarian settings

GBV primary prevention programs seek to facilitate change by addressing the underlying causes and drivers of GBV at a population level. Such programs have traditionally included initiatives to economically empower girls and women, enhanced legal protections for GBV, enshrining women’s rights and gender equality within national legislation and policy, and other measures to promote gender equality. Increasingly, programs are also targeting transformation of social norms that justify and sustain acceptance of GBV. Social norms are contextually and socially derived collective expectations of appropriate behaviors [14]. Families and communities have shared beliefs and unspoken rules that both proscribe and prescribe behaviors that implicitly convey that GBV against women is acceptable, even normal [15, 16]. This includes social norms pertaining to sexual purity, family honor, and men’s authority over women and children in the family. Community leaders, institutions, and service providers, such as health care, education and law enforcement, can reinforce harmful social norms by, for example, blaming women and girls for the sexual assault they experience, or by justifying a husband’s use of physical violence as a means to discipline his wife. Both behaviors are viewed as essential to protect the family’s reputation in the larger community [16].

Diverse academic disciplines have developed different theories to explain the complexity of social norms and their influence on behavior. We use social norms theory as elaborated in social psychology [17]. This theory conceptualizes social norms as beliefs of two types: 1) an individual’s beliefs about what others typically do in a given situation (i.e., descriptive norm); and 2) their beliefs about what others expect them to do in a given situation (i.e., injunctive norm) [18–20]. For this study, we focus on developing a measure of injunctive norms—defined in this case as beliefs about what influential others (e.g., parents, siblings, peers, religious leaders, teachers) expect individuals to do in the case of GBV.

Even with the multiple challenges of humanitarian settings (e.g., separation of families, insecurity and limited resources), there is an opportunity to develop, implement, and evaluate innovations in GBV programming. In such settings, displacement and conflict have created situations where social rules about who can do what necessarily bend to accommodate new realities [16]. Women, for example, may be forced to assume new roles in the family and community, such as having decision-making power and control over household financial resources and assets and working outside the home to help support the family. These changing roles then lead to shifts in behavior and potentially power relations in the family and community that challenge traditional norms around male authority and women’s relegation to the domestic sphere. These circumstances can provide an opportunity to initiate GBV primary prevention efforts, such as those that engage community leaders and members in critical reflection on norms that legitimate gender inequality and what actions can be taken by the individual, family, and community to change norms that cause harm [15, 16]. Acknowledging the potential of the humanitarian setting as an opportunity for primary prevention programming and recognizing the need to strengthen GBV response systems, the United Nations Children’s Fund (UNICEF) built on their work to end female genital mutilation using social norms theory [19] to develop the *Communities Care Program: Transforming Lives and Preventing Violence* Program (Communities Care) [21]. The goal of Communities Care is to create safer communities for women and girls by challenging social norms that sustain GBV and catalyzing new norms that uphold women and girls’ equality, safety, and dignity [15, 21]. The description of the Communities Care program is published elsewhere [15, 16, 21].

However, a significant limitation for evaluating the effectiveness of GBV prevention programs such as Communities Care is the lack of validated instruments to measure change in norms supporting GBV. Therefore, our goal was to create a brief, valid, and reliable measure to examine change over time in harmful social norms and personal beliefs that maintain and tolerate sexual violence and other forms of GBV in low resource and complex humanitarian settings.

While validated instruments exist to measure attitudes towards gender roles and some types of GBV [22, 23], social norms are different from individual attitudes. For nearly two decades, the Demographic and Health Surveys (DHS), which are nationally representative surveys conducted in low and middle-income countries (LMIC), have provided information on attitudes about the acceptability of IPV or wife beating. Respondents are asked whether a man is justified in beating his wife in five different situations: a wife goes out without her husband’s permission; she neglects to keep the children well fed; she argues with her husband in public; she refuses to have sexual intercourse with her husband; and she does not prepare her husband’s meal on time. Response options for these questions are as follows: “agree,” “disagree,” “refuse to answer,” and “don’t know.” These questions are designed specifically to elicit *personal beliefs* (attitudes) about IPV; they have generally functioned well in that they capture various levels of endorsement of IPV both within and among settings, and respondents routinely vary their answers based on the transgression mentioned.

Investigators, however, have raised questions about whether the DHS questions reflect respondents’ own personal beliefs on the acceptability of beating or women’s perception of the social norm operative in their setting. Cognitive interviews with women in Bangladesh, for example, suggested that women’s interpretation of the attitude questions switched between personal and normative beliefs, although it is difficult to know whether this happens routinely in other settings, or whether it was a function of the especially low literacy and female mobility of rural Bangladesh [24, 25].

Scientists have also warned that changing key features of a scenario (e.g., setting, perpetrator, infraction committed, perceived intentionality) can influence measured attitudes and perceived norms on the acceptability of GBV. For example, in Uganda, researchers randomly assigned participants to answer attitude and norm questions on wife beating using three separate wordings [26]. The attitude questions compared the traditional wording of the DHS (whether a man is justified in beating his wife for 5 different infractions) to more contextualized scenarios that depicted the wife’s transgression as either willful or beyond her control. To elicit *norms* related to wife beating, participants were asked about the extent to which they thought other people in their village (reference group) would think the behavior described was justified. Response options for the five questions followed a four-point Likert-type scale: “all or almost all, for example, at least 90% of people in your village,” “more than half but fewer than 90% of people in your village,” “fewer than half but more than 10% of people in your village,” and “very few or none, for example, less than 10% of people in your village.”

The findings demonstrated that when measuring both attitudes and social norms, adding contextual details about the intentionality of a wife’s transgression changed participants’ perception of the acceptability of IPV. In the vignettes, wives who intentionally violated norms about acceptable wifely behavior had a “large” effect [27] on increasing the number of items for which wife beating was viewed as acceptable. In contrast, the vignette that depicted the wife as unintentionally violating norms of behavior had a “small” effect in decreasing the number of items where IPV was considered acceptable. The study authors interpreted this difference as measurement error, arguing that question wordings without context may mis-represent attitudes and norms on violence. While context does matter, the specific details added in this study were likely critical to its findings. Qualitative studies have repeatedly shown that wife beating in LMIC is understood as “discipline” and its acceptability varies depending on the nature of the transgression (whether it is perceived as for “just cause”), who is doing the “correction,” and whether the beating stays within acceptable bounds of severity [24, 25, 28–30].

In this paper, we describe the formative research and psychometric testing of the *Social Norms and Beliefs about Gender Based Violence (GBV) Scale*. The *Scale* is designed to measure change over time in harmful social norms and personal beliefs associated with violence against women and girls among men and women community members in low resource and complex humanitarian settings. The development and validation of the scale was essential for use in measuring change in harmful social norms and beliefs among community members in districts and regions implementing the Communities Care program in two countries with ongoing humanitarian crises, Somalia and South Sudan. The development and testing of the scale was conducted in two phases: 1) formative phase of qualitative inquiry to identify social norms and personal beliefs that sustain and justify GBV perpetration against women and girls across the lifespan in low-resource and humanitarian contexts; and 2) testing phase using quantitative methods to conduct a psychometric evaluation of the new scale in targeted areas of Somalia and South Sudan.

## Methods

### Study settings

The formative and testing phases of the psychometric evaluation was conducted in two countries, Somalia and South Sudan. In Southern Central Somalia, we worked in four districts (Bondhere, Karaan, Wadajir, Yaqshid**)** in Mogadishu and in South Sudan, we worked in two regions (Yei and Warrap). Somalia has experienced more than two decades of conflict as well as ongoing emergencies including drought, famine, and a large number of internally displaced people (IDPs). Yei is located in southwestern South Sudan and was the re-entry point for South Sudanese who fled to the Democratic Republic of Congo (DRC) and Uganda during the Second Sudanese Civil War. Since many people stayed in Yei upon returning, there is conflict between those native to Yei and IDPs from other regions of South Sudan. Warrap is in the northern region of South Sudan and is a gateway between South Sudan and Sudan. Militia activity, cattle-raiding, and conflict over oil, along with the influx of people returning to South Sudan, has caused significant challenges for access to and use of limited resources. The districts and regions in each country were selected based on multiple factors. We focused efforts on districts and regions where GBV reporting systems existed and could be accessed to generate data on case reports and referrals. When engaging GBV survivors and other community members in research on sensitive issues it is essential to have partnerships with diverse service sectors (e.g., health, protection, legal, advocacy) for participants that disclose GBV and request referrals. The evaluation also required safe access to the sites and security while doing the study for both participants and local researchers, therefore this required establishing relationships and obtaining permission from national, regional, and district governmental authorities and ministries as well as traditional leaders in the communities.

### Phase 1: Formative phase methods

For the formative phase, we worked with local partners to identify male and female key stakeholders (e.g., religious leaders, youth and women’s group leaders, advocates for GBV survivors, health providers, child protection staff, police officers, traditional leaders, elders, and teachers) to advance our understanding of and identify harmful and protective social norms associated with GBV within and across settings. The focus group guide was developed and translated to the local language in partnership with team members in each setting. Johns Hopkins provided in-depth training to local staff on facilitating focus groups, data collection, human subjects’ protections, working with distressed participants, and providing referrals to services as appropriate. The focus group guide focused on identification of social norms that protect women and girls from sexual violence and other forms of GBV, norms that are harmful (e.g., hide, sustain, or encourage), norms about disclosing and reporting sexual violence and other forms of GBV to authorities, and who are the people in the family or larger community that are influential in maintaining and changing social norms. For example, the team used scenarios created from aggregating GBV experiences in each setting to explore social norms about the situations and the survivor-perpetrator relationship. We varied the perpetrator and circumstances in each scenario from the perpetrator being a family member, a known person to the family but not part of the family, and an unknown person. For each scenario, focus group participants were asked about their beliefs and norms about how the family and community would respond to victims of the sexual assault or other forms of GBV, if the assault would be reported to authorities, and reasons for reporting or not reporting the assault.

### Qualitative analysis

A qualitative descriptive approach was used to identify themes related to harmful and protective social norms within and across settings. The transcripts were read by three research team members to identify thematic codes. Themes with sub-themes were identified and defined by exemplars or quotes from the transcripts. The three researchers independently assigned codes and discrepancies in coding were discussed in weekly meetings. The codes and corresponding quotes were used to write items for the scale representing each of the identified themes. The themes, sub-themes, and items were then shared with the in-country teams in a joint Somalia/South Sudan meeting. The relevance of the themes and their interpretation for each context was discussed leading to a refinement of the items. Meeting participants from each country rated the importance of each item and offered suggestions on wording of the items to ensure they were capturing the relevant aspects of the different contexts and cultures.

#### Results of phase 1: Formative phase

A total of 42 focus groups (22 in Somalia and 20 in South Sudan) with a total of 215 participants (111 in Somalia and 104 in South Sudan) were conducted. The composition of the focus groups varied by stakeholders (e.g., religious leaders, service providers, teachers, police, youth, elders), age (under 30, 31–45, and 46+), marital status, and sex. Themes identified for social norms that are protective against GBV included parents teaching/guiding children, marriage, and respect for female members of the family. Themes identified as harmful social norms included men’s responsibility/right to correct female behavior and the social expectation that a woman will obey her husband and fulfill her gender prescribed duties to his satisfaction, protecting the family’s dignity by not reporting violence/assault to avoid stigma associated with being a victim, husband’s right to force his wife to have sex, lack of status for women, and forced marriage. Mothers, fathers, parents, community and religious leaders, and male relatives were seen as people that influenced behavior and protected women and girls from GBV. Men and women’s behavior also emerged as subthemes associated with harmful social norms, such as indecent dressing, being out in public alone, and drug/alcohol use. Stigma associated with being a GBV victim, blaming women and girls for the violence/assault, and the importance of family honor and respect were identified as norms that prevent victims and families from reporting sexual violence and other forms of GBV to authorities. Items for the new scale were written for each of the themes and sub-themes relevant to harmful social norms and after elimination of redundant items, 30 items remained and were presented to the in-country teams. After discussion about the focus group themes and the items with the in-country teams, a total of 18 items remained. The team then collaborated to develop introductory statements and response scales for each of two domains of the scale, personal beliefs and injunctive social norms. The final scale to be tested in the evaluation phase had two sets of the 18 items, one for each domain.

#### Methods for phase 2: Psychometric testing

##### Sample

At each of the three sites in the two countries detailed above, trained local research assistants (RAs) recruited and consented 200 community members (15 years and older) to complete the *Social Norms and Beliefs about Gender Based Violence Scale.* The sampling frame was stratified by age group (15–18, 19–24, 25–45, 46+ years) and sex with a target of 25 people per age group/sex combination. As suggested by the in-country teams, male RAs recruited and interviewed male community members and female RAs recruited and interviewed female community members. Each RA recruited participants across age groups. The RA started from a central point determined by the research coordinator each morning. The RA would contact every 3rd house/dwelling counting on both sides of the street/pathway. If nobody was home, the person was not willing to participate, or the person did not match the sampling target for sex/age, the RA went to the next house/dwelling. Once a RA identified and consented an eligible participant in the household and completed the scale, the RA started the process to identify the next eligible participant by going to the next 3rd house/dwelling on the street/pathway. Only one eligible household member completed the scale.

##### Field procedures

RAs received detailed training on protocols for maintaining participant confidentiality and safety as well as protocols designed to ensure safety and security for the team members. In the field, when a RA identified an adult at a house/dwelling, he/she introduced the study. If that person met the eligibility criteria and agreed to participate, the RA worked with the participant to find a private and comfortable place to provide informed consent and administer the scale. If that person did not meet eligibility, he/she was asked if there was someone living in the household that did meet the eligibility. The RA provided each potential participant with informed consent information using the script provided on the study tablet and approved by the in-country team and the Johns Hopkins Medical Institution Institutional Review Board (IRB). If the eligible participant provided verbal consent the RA continued and administered the scale with brief demographic questions, including marital status, employment, and children in the household. The responses were entered by the RA directly on the tablet. Once finished, the RA thanked the participant for their time and answered any questions prior to moving on.

##### Measures

The 18 items generated from the formative phase were asked in two sets to capture the two domains, personal beliefs and injunctive norms. The injunctive social norms items started with “How many of the people whose opinion matters most to you….” with the response scale of: 1 – None of them, 2 – A few of them, 3 – About half of them, 4 – Most of them, and 5 – All of them. The personal beliefs items started with “We would like to know if you think any of the following statements are wrong and should be changed in your community. We also would like to understand how ready or willing you are to take action by speaking out on the issues you think are wrong” and used the response scale: 1 – Agree with this statement, 2 – I am not sure if I agree or disagree with this statement, 3 – I disagree with the statement but am not ready to tell others, and 4 – I disagree with the statement and I am telling others that this is wrong. The scale was translated into Somali and the translation was reviewed by the Somalia team and revised before it was programmed into the study tablet. In South Sudan, the scale was administered in the Kakwa language in Yei and Dinka language in Warrap. As these are not commonly written languages in South Sudan, the team preferred using the English version of the scale programmed on the tablet and translated into the local language at time of administration. The South Sudan team training included discussions and decisions on correct translation of items in the two languages and then the team practiced administering with volunteers not participating in the study to ensure consistency in real-time translation across RAs and sites.

##### Psychometric analyses

For each of the two domains of the scale, we examined construct validity with factor analysis using the common factor model with oblique rotation. Factor loadings of .40 or above were considered as loading on a given factor [31]. Items that did not load on any factor were considered for revision or elimination from the scale. Reliability was estimated with Cronbach’s alpha for each factor subscale. Known groups validity was examined by testing two a priori hypotheses: H_1_: The sites (Somalia, Yei, South Sudan, and Warrup, South Sudan) differ on social norms and personal beliefs due to differences in the extent of GBV programming within the districts of Mogadishu and regions of South Sudan; and H_2_: Men and women participants will differ on social norms and personal beliefs related to GBV. The first hypothesis was tested with analysis of variance and the second with t-tests.

## Results of psychometric testing

The team administered the *Social Norms and Beliefs about GBV Scale* to 602 community members across Mogadishu, Somalia and Yei and Warrup, South Sudan. The sampling frame was successfully implemented by the research team with 50.0% of participants across the settings being female and 50.0% male with an equal distribution across age groups except in Yei, South Sudan. The team in Yei reported having difficulty finding community members in the region over 60 years of age. The lack of older community members could be related to deaths in the Second Civil War from 1983 to 2005. Over half (58.6%) of the participants were married and had children in the home (67.4%). One third (34%) reported working outside the home, 10.1% were looking for work, 21.4% were students, 29.4% were housewives, and 4.7% were too old to work. Table 1 summarizes the characteristics of the participants by country and site.

**Table 1:** Demographic characteristics of participants N (percent)

	Yei *N* = 200	Warrup *N* = 201	Mogadishu *N* = 201
Gender			
Male	100 (50.0)	100 (49.8)	101 (50.2)
Female	100 (50.0)	101 (50.2)	100 (49.8)
Age			
15–17 years old	42 (21.0)	38 (18.9)	47 (23.4)
18–24 years old	54 (27.0)	42 (20.9)	42 (20.9)
25–44 years old	49 (24.5)	41 (20.4)	41 (20.4)
45–60 years old	43 (21.5)	39 (19.4)	39 (19.4)
61 or older	11 (5.5)	40 (19.9)	40 (19.9)
Missing	1 (0.5)	1 (0.5)	0
Marital Status			
Married	107 (53.5)	121 (60.2)	125 (62.2)
Previously married	29 (14.5)	22 (10.9)	21 (10.4)
Never married	64 (32.0)	56 (27.9)	54 (26.9)
Did not respond	0	2 (1.0)	1 (0.5)
Children			
1 or more children	134 (67.0)	138 (68.7)	134 (66.7)
Occupation			
Housewife	50 (25.0)	55 (27.4)	72 (35.8)
Working	66 (33.0)	67 (33.3)	74 (36.8)
Looking for work	22 (11.0)	17 (8.5)	22 (10.9)
Too old to work	9 (4.5)	13 (6.5)	6 (3.0)
Student	53 (26.5)	49 (24.0)	27 (13.4)

### Factor analysis

The factor analysis for the items in the injunctive norms domain of the scale was based on responses from participants that completed all items (*N* = 587, 97.5%). There were 3 of the 18 items on the injunctive social norms scales that did not load on any factor and were thus removed from the scale. The first item “expect daughters to be married before 15 years of age” likely did not correlate with the other items on the scale because early marriage is seen as a different concept than sexual violence. The second item “think that if an unmarried woman/girl is raped by a man, she should marry him rather than not being married at all” captures two different concepts—marrying the man who raped her and that being better than not being married at all. This complexity likely made the question difficult to answer. The third item “expect a woman not to report her husband for forcing her to have sexual intercourse” did not reflect a consistent social norm. Discussions with the in-country teams revealed that there was considerable debate on this item even among people who agreed on other items. Based on the eigenvalues (first 5 eigenvalues were 4.27, 1.82, 1.23, 0.94, 0.81), the remaining 15 items formed three factors (Table 2 presents the factor loadings for each item on each of the three factors) with each item loading above 0.40 on only one factor. The following titles were given to represent the three factors, later describes as subscales: “Response to Sexual Violence” has 5 items, “Protecting Family Honor” has 6 items, and “Husband’s Right to Use Violence” has 4 items. The “Response to Sexual Violence” and “Husbands’ Right to Use Violence” subscales had the highest inter-factor correlation (0.46) followed by “Response to Sexual Violence” and “Protecting Family Honor” (0.34), then “Protecting Family Honor” and “Husbands’ Right to Use Violence” (0.30). Importantly, these 3 factors were consistent with and reflected the themes identified from the qualitative analyses of the focus groups in Phase 1. A very similar factor structure was found for the personal beliefs domain (*N* = 588, 97.7%). Eigenvalues (first 5 eigenvalues were 4.46, 1.76, 1.46, 0.90, 0.88) suggested 3 factors as illustrated in Table 3. All items loaded at 0.45 or greater on only one of the three factors. One item, “a woman/girl would be stigmatized if she were to report rape” loaded on the “Response to Sexual Violence” in the personal beliefs domain whereas the corresponding item, “women/girls fear stigma if they were to report sexual violence”, loaded on the “Protecting Family Honor” subscale for the social norms domain. The inter-factor correlations on the personal beliefs domain were also very similar to the injunctive social norms domain scale: “Response to Sexual Violence” and “Husbands’ Right to Use Violence” had the highest correlation (0.43) followed by “Response to Sexual Violence” and “Protecting Family Honor” (0.32), then “Protecting Family Honor” and “Husbands’ Right to Use Violence” (0.26).

**Table 2:** Factor loadings and Cronbach alphas (last row of table) for the injunctive social norms scales (*N* = 587)

How many of the people whose opinion matters most to you:	Response to Sexual Violence	Protecting Family Honor	Husband’s Right to Use Violence
Expect a husband to abandon his wife if she reports that she has been raped	.671	.038	.020
Expect the family to ignore/reject a daughter if she reports that she has been raped	.556	.100	.023
Accept sexual violence against women and girls a normal part of life	.507	.091	.159
Blame women/girls when they are raped	.477	.054	.141
Think that a man should have the right to demand sex from a woman or girl even if he is not married to her	.476	−.014	.146
Expect women/girls to not report rape to protect the family dignity	−.026	.739	−.092
Expect that a woman/girl’s reputation will be damaged if she reports sexual violence to the authorities or elders	.136	.594	.030
Fear stigma if they were to report sexual violence	.212	.522	−.097
Expect sexual violence to be handled within the family and not reported to authorities	.186	.521	−.033
Expect a husband or father to retaliate against the alleged perpetrators	−.207	.445	.148
Expect women and girls to only report sexual violence if they have serious physical injuries	.055	.419	.049
Think that when a man beats his wife, he is showing his love for her	−.025	−.015	.662
Think that a man has the right to beat/punish his wife	.129	.030	.618
Think it is okay for a husband to beat his wife to discipline her	.040	.075	.552
Expect a husband to force his wife to have sex when she does not want to	.143	−.061	.430
Cronbach’s Alpha	0.75	0.73	0.69

**Table 3:** Factor structure and Cronbach’s alpha (last row of table) for the personal beliefs scales (*N* = 587)

How ready or willing are you to take action by speaking out on each issue	Response to Sexual Violence	Husband’s Right to Use Violence	Protecting Family Honor
Husbands should abandon/reject/divorce their wife if she reports that she has been raped	.616	−.016	−.075
A man should have the right to demand sex from a woman or girl even if he is not married to her	.537	.113	.092
A woman/girl would be stigmatized if she were to report sexual violence	.524	−.275	−.326
A woman/girl should be blamed when she has been raped	.506	.137	.024
Sexual violence against women and girls should be accepted as a normal part of life	.457	.127	−.070
Families should ignore/reject a daughter if she reports that she has been raped	.454	.027	−.017
It is okay for a husband to beat his wife to discipline her	.034	.707	−.095
When a man beats his wife, he is showing his love for her	.037	.635	−.025
A man has the right to beat/punish his wife	.240	.580	−.059
A husband should force his wife to have sex when she does not want to	.192	.464	−.045
Women/girls should not report rape to protect the family dignity	.000	.028	−.714
A woman/girl’s reputation will be damaged if she reports sexual violence to the authorities	.133	.003	−.641
Sexual violence should be handled within the family and not reported to authorities	.132	−.127	−.621
A husband or father should retaliate against the alleged perpetrators	−.144	.276	−.512
Women and girls should only report sexual violence if they have serious physical injuries	−.016	.053	−.497
Cronbach’s Alpha	0.71	0.77	0.75

### Reliability

Cronbach alpha reliabilities, a measure of internal consistency of the scale, were in an acceptable range for all factors/subscales within each domain. Cronbach alphas ranged from 0.69 to 0.75 for the injunctive norms domain and 0.71 to 0.77 for the personal beliefs domain (the last row of Tables 2 and 3 present the Cronbach alphas for each scale).

### Descriptive statistics

Scores for each of the factors (subscales) were computed by taking the average of the items within the subscales. The injunctive social norms domain subscales scores range from 1 to 5 with higher scores reflecting more negative responses to sexual violence and GBV, stronger support for social norms that prioritize protecting family honor by not reporting sexual violence or other forms of GBV, and stronger support for norms endorsing a husband’s right to use violence. Personal beliefs subscales can range from 1 to 4 with higher scores reflecting a more positive response to survivors of sexual violence, that protecting family honor and not reporting sexual violence is wrong, and that a husband should not have the right to use violence against his wife. The means, standard deviations, minimum, and maximum observed score for each of the subscales in each domain are presented in Table 4. In general, the mean for the injunctive social norms subscales reflect participants’ views that “few to about half” of the people who are important/influential to them endorse harmful social norms about GBV with “Protecting Family Honor” being the strongest norm (means range from 2.00 to 2.77). The mean for the personal beliefs subscales reflects that participant beliefs range between “not being sure if they disagree” with the norms to “disagreeing but not being ready to speak out against them.” Specifically, participants’ beliefs ranged between not being sure if they disagree to disagreeing but not ready to speak out against protecting family honor (mean = 2.61) and husband’s right to use violence (mean = 2.90). Participants indicated that they were between disagreeing but not being ready to tell others to telling others that negative responses to sexual violence survivors are wrong (mean = 3.29). Cross domain correlations were − .318 (p < .001) for “Response to Sexual Violence”, −.512 (p < .001) for “Protecting Family Honor”, and − .427 (p < .001) for “Husband’s Right to Use Violence.”

**Table 4:** Descriptive statistics for subscales within each domain (*N* = 587)

	Min	Max	Mean	Std. Dev.
Injunctive Social Norms^1^				
Response to Sexual Violence	1.00	4.60	2.00	0.77
Protecting Family Honor	1.00	5.00	2.77	0.79
Husband’s Right to Use Violence	1.00	5.00	2.38	0.84
Personal Beliefs^2^				
Response to Sexual Violence	1.33	4.00	3.29	0.64
Protecting Family Honor	1.00	4.00	2.61	0.88
Husband’s Right to Use Violence	1.00	4.00	2.90	0.93

^1^Social Norm Response Scale: 1 – None of them, 2 – A few of them, 3 – About half of them, 4 – Most of them, 5 – All of them

^2^Personal Beliefs Response Scale: 1 – Agree with this statement, 2 – I am not sure if I agree or disagree with this statement, 3 – I disagree but am not ready to tell others, 4 – I am telling others that this is wrong

### Known groups validity

Analysis of variance with Bonferroni post-hoc tests revealed that the three sites differed significantly on all subscales for the injunctive social norms domain (i.e., “Response to Sexual Violence,” p < .001; “Protecting Family Honor,” p = .039; “Husband’s Right to Use Violence,” p < .001). Women and men participants in Yei, South Sudan, where there are few GBV programs and services, reported social norms that are significantly more accepting of sexual violence and other forms of GBV than Warrap, South Sudan and Mogadishu, Somalia. In terms of personal beliefs, women and men in Yei were also significantly less likely to speak out against harmful responses to sexual violence and other GBV (p < .001). In Mogadishu, Somalia, men and women were significantly less likely to speak out against “Protecting Family Honor” (p < .001) and “Husband’s Right to Use Violence” (p < .001) than the sites in South Sudan. Table 5 summarizes the t-test results examining differences in the subscales for both domains between men and women. Women participants had significantly higher scores on all of the subscales for the injunctive social norms, indicating women were more likely to endorse harmful norms related to “Response to Sexual Violence”, “Protecting Family Honor”, and “Husband’s Right to Use Violence” than men. Men and women did not differ on personal beliefs about “Response to Sexual Violence”, however, men reported that they are more ready to speak out against harmful social norms of “Protecting Family Honor” and “Husband’s Right to Use Violence” than women.

**Table 5:** Gender differences on subscales within the Injunctive Social Norms and Personal Beliefs domains – Means (SD)

	Male *N* = 297	Female *N* = 299	*p*-value
Injunctive Social Norms			
Response to Sexual Violence	1.90 (0.64)	2.10 (0.88)	.002
Protecting Family Honour	2.67 (0.78)	2.88 (0.80)	.001
Husband’s Right to Use Violence	2.26 (0.71)	2.50 (0.94)	<.001
Personal Beliefs			
Response to Sexual Violence	3.32 (0.67)	3.26 (0.59)	.250
Protecting Family Honour	2.77 (0.96)	2.45 (0.86)	<.001
Husband’s Right to Use Violence	3.02 (0.89)	2.78 (0.91)	.001

## Discussion

The psychometric properties of the *Social Norms and Beliefs about GBV Scale* (final scale is presented in Additional file 1) are strong. Each of the three subscales, “Response to Sexual Violence,” “Protecting Family Honor,” and “Husband’s Right to Use Violence” within the two domains of the scale illustrate good factor structure, acceptable internal consistency, reliability, and are supported by the significance of the hypothesized group differences. These three factors represent social norms that are known from previous research to maintain the high rates of GBV in many global settings [28]. The “Response to Sexual Violence” subscale captures the individual, family, and community response of blaming the victim for GBV. Most often a woman or girl is blamed for the sexual assault or other form of GBV and the family and larger community can respond with rejection and judgement of her behavior, which can result in the family not supporting or abandoning the victim. It reflects the acceptance of sexual violence and other forms of GBV as expected or even normal and that women and girls need to limit their movement and actions to prevent men from assaulting them, as men are not able to control their behavior if they are “tempted” by women. High scores on the injunctive norms domain of this subscale represent that the respondents believe that their influential others expect people to endorse victim blaming responses to sexual violence and other forms of GBV. The “Protecting Family Honor” subscale identifies the stigma associated with being a member of a family/clan where a women/girl experiences GBV and the importance placed on addressing the violence within the family/clan rather than reporting it to authorities. The priority is to protect the family and victim’s reputations rather than the safety and well-being of the woman or girl. High scores on the injunctive domain of this subscale represent that the respondent believes their influential other expects people to prioritize protecting family honor over safety and well-being of victims. The “Husband’s Right to Use Violence” subscale reflects social norms that support a husband’s use of violence to discipline his wife and to have sex with her even when she does not want to. It also reflects a norm that associates a man’s use of violence against his wife with illustrating his love for her. High scores on the injunctive norms domain for this subscale indicates that the respondents believe their influential others expect people to endorse a husband’s right to use violence against his wife. High scores on the personal beliefs domains for each of the subscales reflect a greater willingness to speak out against social norms that endorse GBV.

Validity of the injunctive norms subscales was supported by significant relationships with other variables (i.e., site and sex) as hypothesized during the development of the scale. The three sites were significantly different on the injunctive norms domain of the scale. Although all three sites experienced a high degree of conflict, the amount of humanitarian services to support GBV survivors and programming to raise awareness and change harmful social norms towards GBV varied. Mogadishu districts participating in the study had relatively active programming, with Warrap and Yei reporting few international and local NGOs with capacity to provide diverse GBV services and programs. Yei, South Sudan was found to have significantly stronger norms that endorse negative “Response to Sexual Violence” and other forms of GBV than other sites. The beliefs of participants from Yei also indicated less support for changing harmful social norms about GBV than other sites in the study. Participants in the four districts of Mogadishu scored the lowest on the personal beliefs subscales of “Husband’s Right to Use Violence” and “Protecting Family Honor.” This finding indicates that participants were less willing to speak out against social norms that support husbands’ rights to use violence against their wives or norms that support not reporting sexual violence to protect family honor than the South Sudan sites. Important to interpreting the findings are the differences in context, culture, and religion across the sites which inform social norms and personal beliefs.

Generalizability is one of the indicators of trustworthiness of the *Social Norms and Beliefs about GBV scale* – the ability to interpret and apply the scale in a broader context to make it relevant and meaningful to GBV prevention programs being implemented and evaluated in diverse low-resource and humanitarian settings. Importantly, the 36-item two domain scaled applied with community members by local teams in diverse districts and regions within Somalia and South Sudan resulted in a valid and reliable 30-item scale to measure personal beliefs and injunctive social norms. The psychometric phase included randomly selected women and men across multiple age groups (15 years and older), living in both urban and rural communities, and included community members living in settlements and camps for displaced persons. Thus, the scale has the potential to be used in not only humanitarian settings, but also GBV prevention programs in other low-resource and fragile settings.

Although this psychometric evaluation has several strengths, including a mixed methods design to develop the scale and a large sample size to test the scale across diverse sites, it has limitations. The study does not include a separate validation sample to conduct a confirmatory factor analysis. Further, we did not test the relationship between the *Social Norms and Beliefs about GBV Scale* and community members’ reports on experience, perpetration, or witnessing of GBV in the participating communities. The research team decided in collaboration with local partners not to ask participants in the evaluation phase about personal experiences with GBV for either the scale development or testing. The local colleagues felt community members would be more comfortable and likely to participate in the scale development and testing if they were not asked about their own experiences and thus also increasing generalizability.

## Conclusion

The study presents a mixed methods approach to developing a brief scale with strong psychometric properties to measure change in harmful social norms associated with GBV. The *Social Norms and Beliefs About GBV Scale* is a 30-item scale with three subscales, “Response to Sexual Violence,” “Protecting Family Honor,” and “Husband’s Right to Use Violence” in each of the two domains, personal beliefs and injunctive social norms. The scale to our knowledge is one of the first to demonstrate good factor structure, acceptable internal consistency, and reliability, and be supported by the significance of the hypothesized group differences by setting and sex. We encourage and recommend that researchers apply the *Social Norms and Beliefs about GBV Scale* in different humanitarian and global LMIC settings and collect parallel data on a range of GBV outcomes. This will allow us to further validate the scale by triangulating its findings with GBV experiences and perpetration and assess its generalizability across diverse settings.

## Additional file

Additional file 1: Social Norms and Beliefs about Gender Based Violence Scale. (DOCX 17 kb)

## Abbreviations

DHS: Demographic and Health Surveys
DRC: Democratic Republic of Congo
GBV: Gender-based violence
IASC: Inter-Agency Standing Committee
IDP: Internally displaced persons
IPV: Intimate partner violence
IRB: Institutional Review Board
LMIC: Low and middle-income countries
NPV: Non-partner violence
RA: Research assistant
UNICEF: United Nations Children’s Fund
