# Supplementary material for: Social norms and beliefs about gender based violence scale: a measure for use with gender based violence prevention programs in low-resource and humanitarian settings
Source: Confl Health. 2019 Mar 8;13:6. doi: 10.1186/s13031-019-0189-x (PMC6408811; doi:10.1186/s13031-019-0189-x)
Supplement: Supplementary file 1 — Social Norms and Beliefs about Gender Based Violence Scale. (DOCX 17 kb) [file 13031_2019_189_MOESM1_ESM.docx]

**Social Norms and Beliefs about Gender-Based Violence (GBV) Scale**

| Injunctive Norms: For each of the following questions, please tell us how many people whose opinion matters most to you: | | | | | |
| --- | --- | --- | --- | --- | --- |
|  | None of them | Few of them | About ½ of them | Most of them | All of them |
| 1. Expect a husband to abandon his wife if she reports that she has been raped | 1 | 2 | 3 | 4 | 5 |
| 2. Expect the family to ignore/reject a daughter if she reports that she has been raped | 1 | 2 | 3 | 4 | 5 |
| 3. Accept sexual violence against women and girls a normal part of life | 1 | 2 | 3 | 4 | 5 |
| 4. Blame women/girls when they are raped | 1 | 2 | 3 | 4 | 5 |
| 5. Think that a man should have the right to demand sex from a woman or girl even if he is not married to her | 1 | 2 | 3 | 4 | 5 |
| 6. Expect women/girls to not report rape to protect the family dignity | 1 | 2 | 3 | 4 | 5 |
| 7. Expect that a woman/girl's reputation will be damaged, if she reports sexual violence to the authorities or elders | 1 | 2 | 3 | 4 | 5 |
| 8. Fear stigma if they were to report sexual violence | 1 | 2 | 3 | 4 | 5 |
| 9. Expect sexual violence to be handled within the family and not reported to authorities | 1 | 2 | 3 | 4 | 5 |
| 10. Expect a husband or father to retaliate against the alleged perpetrators | 1 | 2 | 3 | 4 | 5 |
| 11. Expect women and girls to only report sexual violence if they have serious physical injuries | 1 | 2 | 3 | 4 | 5 |
| 12. Think that when a man beats his wife, he is showing his love for her | 1 | 2 | 3 | 4 | 5 |
| 13. Think that a man has the right to beat/punish his wife | 1 | 2 | 3 | 4 | 5 |
| 14. Think it is okay for a husband to beat his wife to discipline her | 1 | 2 | 3 | 4 | 5 |
| 15. Expect a husband to force his wife to have sex when she does not want to | 1 | 2 | 3 | 4 | 5 |

| Personal Beliefs: For each of the following questions, please tell us the extent to which you agree or disagree with the statement and your willingness to tell others about your belief | | | | |
| --- | --- | --- | --- | --- |
|  | Agree with this statement | Not sure if I agree or disagree | I disagree but am not ready to tell others | I disagree and am telling others |
| 16. Husbands should abandon/reject/divorce their wife if she reports that she has been raped | 1 | 2 | 3 | 4 |
| 17. A man should have the right to demand sex from a woman or girl even if he is not married to her | 1 | 2 | 3 | 4 |
| 18. A woman/girl would be stigmatized if she were to report sexual violence | 1 | 2 | 3 | 4 |
| 19. A woman/girl should be blamed when she has been raped | 1 | 2 | 3 | 4 |
| 20. Sexual violence against women and girls should be accepted as a normal part of life | 1 | 2 | 3 | 4 |
| 21. Families should ignore/reject a daughter if she reports that she has been raped | 1 | 2 | 3 | 4 |
| 22. Women/girls should not report rape to protect the family dignity | 1 | 2 | 3 | 4 |
| 23. A woman/girl's reputation will be damaged if she reports sexual violence to the authorities | 1 | 2 | 3 | 4 |
| 24. Sexual violence should be handled within the family and not reported to authorities | 1 | 2 | 3 | 4 |
| 25. A husband or father should retaliate against the alleged perpetrators | 1 | 2 | 3 | 4 |
| 26. Women and girls should only report sexual violence if they have serious physical injuries | 1 | 2 | 3 | 4 |
| 27. When a man beats his wife, he is showing his love for her | 1 | 2 | 3 | 4 |
| 28. A man has the right to beat/punish his wife | 1 | 2 | 3 | 4 |
| 29. It is okay for a husband to beat his wife to discipline her | 1 | 2 | 3 | 4 |
| 30. A husband should force his wife to have sex when she does not want to | 1 | 2 | 3 | 4 |

Scoring Instructions: Create three subscales for social norms and three subscales for personal beliefs by taking the mean of the items within each subscale.

Response to Sexual Violence Social Norm (items 1-5)

Protecting Family Honor Social Norm (items 6-11)

Husband’s Right to Use Violence Social Norm (items 12-15)

Response to Sexual Violence Personal Belief (items 16-21)

Protecting Family Honor Personal Belief (items 22-26)

Husband’s Right to Use Violence Personal Belief (items 27-30)
